# Deletion of the *sec4* Homolog *srgA* from *Aspergillus fumigatus* Is Associated with an Impaired Stress Response, Attenuated Virulence and Phenotypic Heterogeneity

**DOI:** 10.1371/journal.pone.0066741

**Published:** 2013-06-13

**Authors:** Margaret V. Powers-Fletcher, Xizhi Feng, Karthik Krishnan, David S. Askew

**Affiliations:** Department of Pathology & Laboratory Medicine, University of Cincinnati College of Medicine, Cincinnati, Ohio, United States of America; Geisel School of Medicine at Dartmouth, United States of America

## Abstract

Small GTPases of the Rab family are master regulators of membrane trafficking, responsible for coordinating the sorting, packaging and delivery of membrane-bound vesicles to specific sites within eukaryotic cells. The contribution of these proteins to the biology of the human pathogenic fungus *Aspergillus fumigatus* has not been explored. In this study, we characterized the *srgA* gene, encoding a Rab GTPase closely related to Sec4. We found that a GFP-SrgA fusion protein accumulated preferentially at hyphal tips and mature condiophores. The radial growth of a Δ*srgA* mutant was impaired on both rich and minimal medium, consistent with a role for SrgA in filamentous growth. In addition, the Δ*srgA* mutant revealed dysmorphic conidiophores that produced conidia with heterogeneous morphology. The Δ*srgA* mutant was hypersensitive to brefeldin A-mediated inhibition of vesicular trafficking and showed increased temperature sensitivity relative to wild type *A. fumigatus*. However, the most striking phenotype of this mutant was its phenotypic heterogeneity. Individual colonies isolated from the original Δ*srgA* mutant showed variable morphology with colony sectoring. In addition, each isolate of the Δ*srgA* mutant displayed divergent phenotypes with respect to thermotolerance, *in vitro* stress response and virulence in a *Galleria mellonella* infection model. Taken together, these results indicate that SrgA contributes to the asexual development and filamentous growth of *A. fumigatus*. However, the discordant phenotypes observed among individual isolates of the Δ*srgA* mutant suggest that the absence of *srgA* exerts selective pressure for the acquisition of compensatory changes, such as second-site suppressor mutations.

## Introduction

Filamentous fungi elongate and branch by apical extension, a mode of growth that involves the establishment of a stable axis of polarity, followed by the maintenance of growth in the same direction [1]. The ability to sustain polarization requires a constant stream of new cell wall and plasma membrane material to the hyphal apex [2]. This is accomplished by packaging components required for membrane and cell wall biogenesis into membrane-enclosed vesicles of the secretory system and delivering them to the growing tip cell [3]. The secretory pathway is also exploited for the transport of hydrolytic enzymes to the hyphal apex, where they are exocytosed into the surrounding substrate to assist with nutrient acquisition [4], [5]. Current evidence suggests that both exocytosis and cell growth are concentrated at the hyphal tips of filamentous fungi, although not exclusively [6]. The Spitzenkörper is an apical cluster of vesicles and cytoskeletal components that assists in this process by providing a vesicle supply center for the rapid delivery of enzymes into and across the apical cell membrane [7]. This contrasts the budding yeast *Saccharomyces cerevisiae*, where the continual delivery of vesicles across the entire cell surface promotes spherical rather than polarized growth [8].

Members of the Rab family of GTPases have pivotal functions in the regulation of vesicular trafficking in eukaryotes. By cycling between inactive (GDP-bound) and active (GTP-bound) states the Rab GTPases, in coordination with their many effector proteins, are able to orchestrate precise spatial targeting of secretory vesicles [9]. The Rab GTPase Sec4 is central to this process, contributing to the transport of vesicles from the trans-Golgi to the plasma membrane [10]. Loss of *sec4* results in the accumulation of secretory vesicles and disruption of protein secretion, which is incompatible with viability in a number of fungal species [10], [11], [12], [13], [14]. Additionally, other Sec4 homologues have been linked to functions that contribute to fungal pathogenesis, such as the formation of specialized infection structures [15] or the extracellular release of vesicles containing virulence-related factors [13].

Very little is known about Rab GTPases in *Aspergillus fumigatus*, an opportunistic human mold pathogen that causes a life-threatening infection known as invasive aspergillosis [16]. In this study, we characterized the *A. fumigatus srgA* gene, encoding a Sec4 homolog that was initially annotated in *Aspergillus niger* as secretion-related GTPase A (SrgA) [17]. An *A. fumigatus ΔsrgA* mutant was constructed and shown to be associated with abnormal colony morphology, attenuated conidiation, reduced hyphal growth, and hypersensitivity to environmental stress. However, there was surprising phenotypic heterogeneity among independent isolates of this mutant with respect to *in vitro* phenotypes and virulence, suggesting that the consequences of losing SrgA function is modified by the activation of different compensatory responses.

## Results

### Identification of the Sec4 Homolog SrgA in *A. fumigatus*

**Figure 1:**
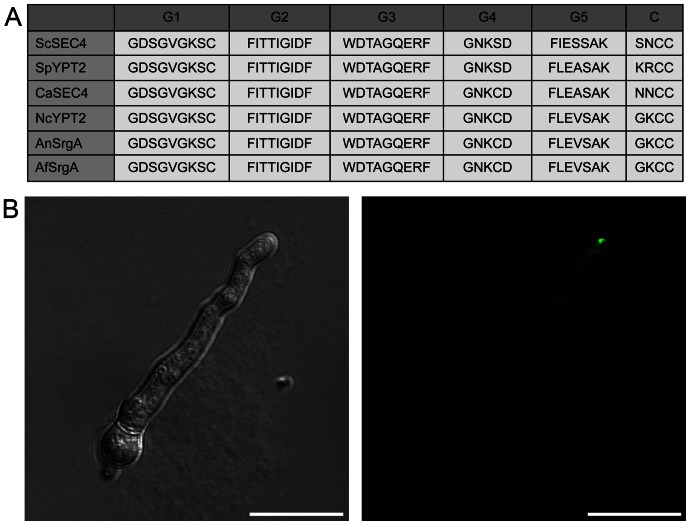
Relationship between *A. fumigatus* SrgA and Sec4 homologs. A: Comparison of G-box motifs (G1–G5) and C-termini (C) from fungal Sec4 homologs in *Saccharomyces cerevisiae (Sc)*, *Schizosaccharomyces pombe (Sp)*, *Candida albicans (Ca*), *Neurospora crassa (Nc)*), *Aspergillus niger (An)*, and *Aspergillus fumigatus (Af)*. B: Intracellular localization of *A. fumigatus* SrgA. The SrgA protein was tagged at its N-terminus with GFP and expressed in *A. fumigatus* under the control of the *gpdA* promoter. Scale bar  = 10 µm.

SrgA was previously identified in *A. niger* as one of five different secretion-related GTPases thought to be involved in mediating different stages of vesicle transport [17]. The corresponding gene in *A. fumigatus* (AFUA_4G04810), encodes a 206 amino acid protein in which multiple Rab-family motifs are found. Included within these shared motifs are the five “G box” sequences, which are present in all small GTPase families [18]. As shown in Figure 1A, there is high sequence homology within these G box motifs between *A. fumigatus* SrgA and other previously characterized fungal Sec4 proteins. Conservation within the G2 domain is particularly noteworthy, as this region is the effector domain, responsible for functional specificity within the Rab GTPase family [17]. Also contributing to Rab GTPase function are two conserved C-terminal cysteine residues, which are posttranslationally modified to allow for, and stabilize, the protein's association with vesicle membranes [19].

To determine the intracellular localization of SrgA, the protein was tagged at its N-terminus with green fluorescent protein (GFP) and expressed in wild type (wt) *A. fumigatus* under the control of the *gpdA* promoter. As shown in Figure 1B, the GFP-SrgA fusion protein accumulated preferentially at hyphal tips, similar to what has been described for Sec4 and related Sec proteins in *Candida albicans* [20], [21]. This localization is consistent with the putative role for SrgA in the regulation of apical vesicle transport in filamentous fungi.

### Loss of SrgA Generates Phenotypic Heterogeneity in Colony Morphology

**Figure 2:**
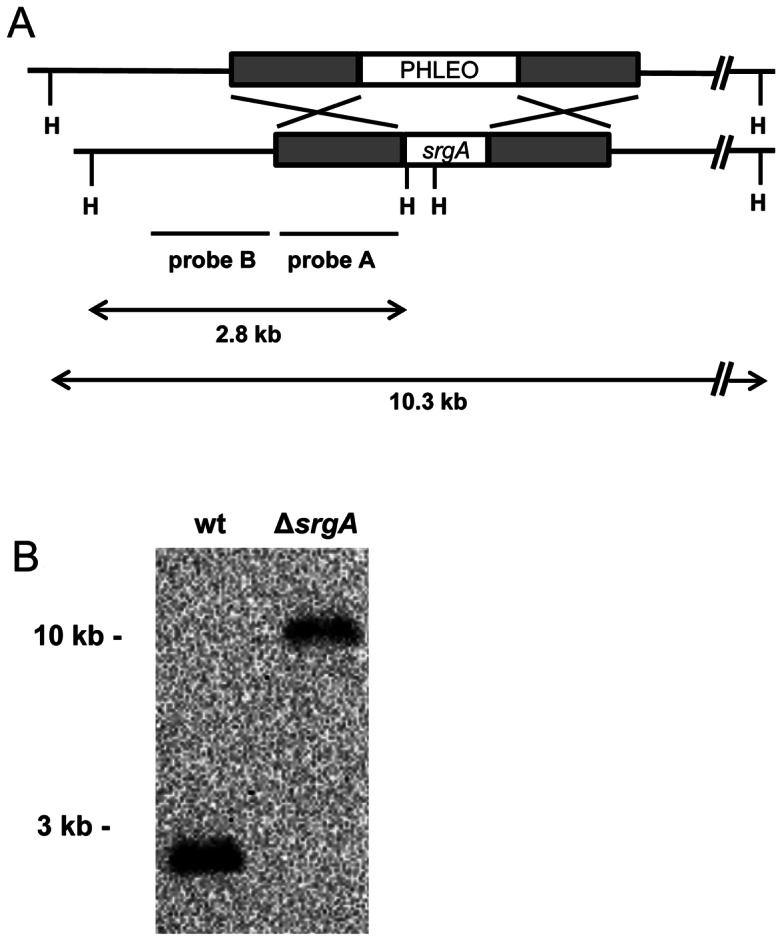
Deletion of *srgA* from *A. fumigatus*. Southern blot analysis of *Hind*III-digested genomic DNA using a probe located upstream of the *srgA* coding region (probe A) identified the predicted 2.8 kb fragment in wt *A. fumigatus*, which was lengthened to 10.3 kb in the Δ*srgA* mutant due to replacement of *srgA* with the phleomycin-resistance cassette (PHLEO).

A Δ*srgA* strain was constructed by replacing the entire *srgA* coding region with a phleomycin-resistance cassette. The expected deletion was identified by probing *Hind*III-digested genomic DNA with a *srgA* 5′ flanking probe (probe A, Figure 2), revealing the loss of the wt 2.8 kb *Hind*III fragment and the appearance of the expected 10.3 kb fragment.

**Figure 3:**
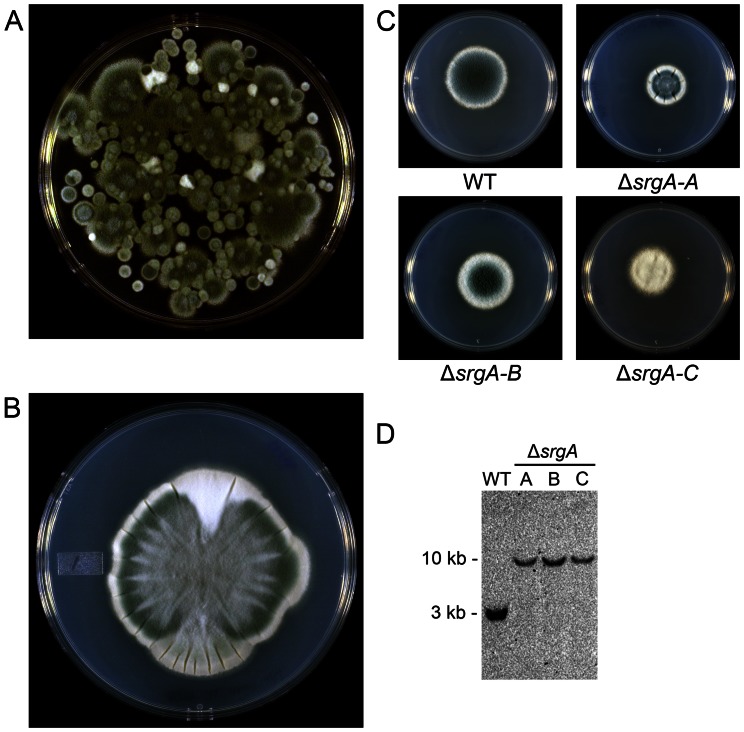
Loss of SrgA is associated with diverse colony morphology. A: Conidia harvested from the initial monoconidial Δ*srgA* mutant produced a heterogeneous population of colonies when spread for isolation. B: Colony sectoring was observed in Δ*srgA* isolates (shown here, isolate-A). C: Three individual Δ*srgA* isolates (A–C) were selected from the heterogeneous population shown in panel A. D: Southern blot analysis of *Hind*III-digested genomic DNA using a probe located upstream of the *srgA* coding region (Figure 2, probe B) identified the predicted 2.8 kb fragment in wt *A. fumigatus*, which was lengthened to 10.3 kb in the Δ*srgA* isolates due to replacement of *srgA* with the phleomycin-resistance cassette.

The Δ*srgA* mutant showed surprising phenotypic heterogeneity when plated for isolation on solid media, manifested by differences in colony size, the level of conidiation and colony sectoring (Figure 3A and 3B). Three distinct colonial morphologies were arbitrarily selected for further phenotypic analysis, using size and conidiation as a crude measure of individuality, hereafter referred to as Δ*srgA* isolates A, B, and C (Figure 3C). Genotypic analysis by Southern blot, using a probe that is upstream of the *srgA* open-reading frame (probe B, Figure 2) confirmed that each Δ*srgA* isolate lacked the *srgA* gene (Figure 3D). Moreover, no wt conidia were recovered by plating the mutant onto non-selective media, suggesting that the mutants are not heterokaryons that are protected by a small population of wt nuclei. The presence of the phleomycin resistance cassette, in the absence of any detectable *srgA* gene was also confirmed by PCR in each of the Δ*srgA* isolates (data not shown). Together, these findings suggest that deletion of *srgA* generates phenotypic diversity in colony morphology, possibly due to the activation of compensatory changes that were selected for based on their ability to improve fitness.

### Loss of SrgA Impairs Conidiation

**Figure 4:**
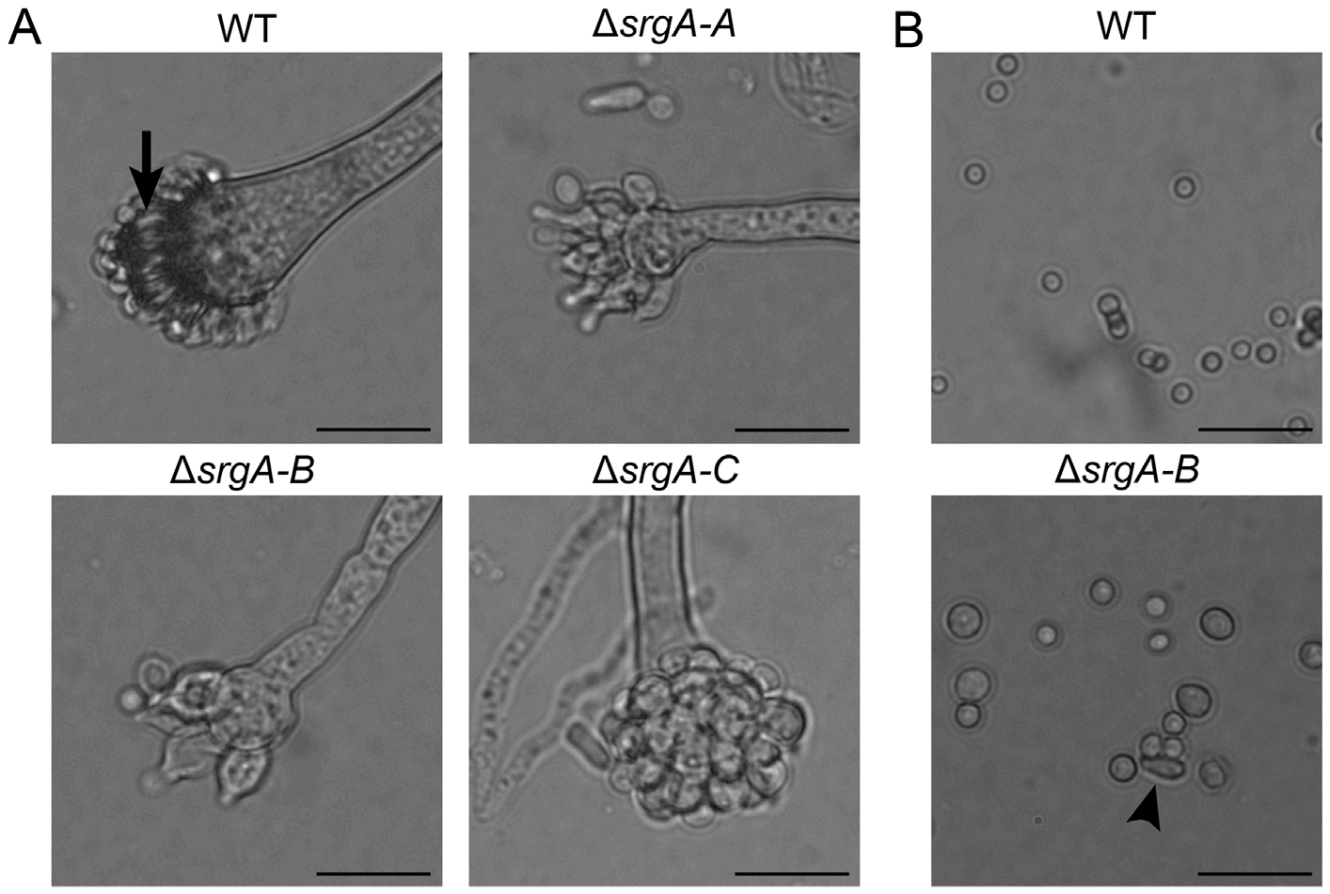
Loss of SrgA impairs conidiation. A: All three Δ*srgA* isolates have attenuated conidophores and dysmorphic phialides (normal phialides are shown by the arrow in wt). B: All three Δ*srgA* isolates release conidia that are heterogeneous in both size and shape. Some of the elongated conidia may be abnormal phialides that are released along with the conidia (arrow) (Scale bar  = 20 µm).

The decreased pigmentation of all Δ*srgA* colonies suggested that loss of SrgA reduces the efficiency of asexual development. Consistent with this, dysmorphic conidiophores were observed in all three of the Δ*srgA* mutant isolates; the vesicle was attenuated in size and the phialides were irregularly shaped, often appearing swollen at the base (Figure 4A). In contrast to wt conidia, which formed uniform spheres approximately 2 µm in diameter, the conidia that were released from Δ*srgA* colonies were heterogeneous in size, ranging from 2–5 µm in diameter (Figure 4B). In addition, all three mutant isolates produced oval and tear-drop shaped conidia, some of which may represent the abnormal release of phialides from the mutant conidiophores rather than true conidia (Figure 4B, arrow). Despite this aberrant morphology, all of the mutant conidia were viable and germinated normally in liquid culture (data not shown). The abnormal conidiation observed in Δ*srgA* colonies could not be rescued by osmotic stabilization of the medium with sorbitol (data not shown). Moreover, none of the Δ*srgA* isolates showed increased sensitivity to the cell wall-targeting antifungal agent, caspofungin (data not shown), suggesting that the reduced conidiation is not due to a major defect in cell wall integrity.

**Figure 5:**
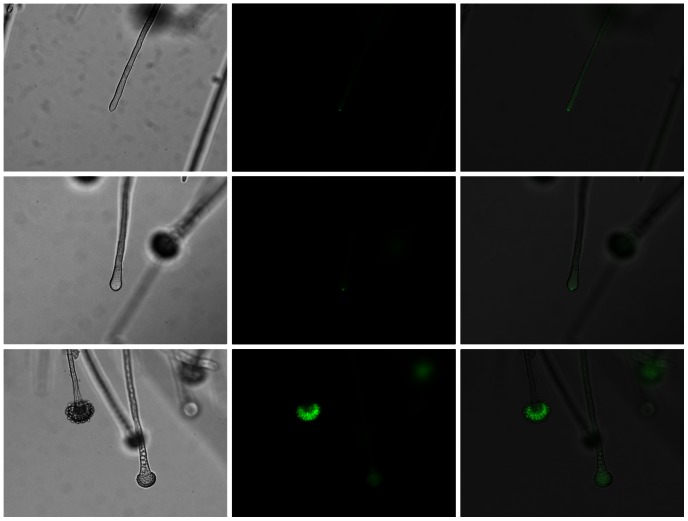
GFP-SrgA localizes to conidiophores. GFP-SrgA localizes to the apex of both hyphae and conidiophores. A punctate accumulation at the tip is seen in both hyphae and the early stages of vesicle swelling (top and middle rows, respectively), but a more diffuse localization is evident in mature conidiophores (bottom row). Left column: brightfield; middle column: GFP fluorescence; bottom column: Merged image.

The aberrant conidiophores in the Δ*srgA* mutant suggested that SrgA may be localized at the site of conidia production. This was confirmed by analysis of GFP-SrgA localization during sporulation. As shown in Figure 5, the GFP-SrgA fusion protein localized to a distinct spot at the apex of young developing conidiophores, which progressively expanded to include the entire vesicle in mature conidiophores. Taken together, these findings suggest that SrgA plays a role in the developmental program, presumably by maximizing the efficiency of vesicle delivery to the developing condiophore.

### Loss of SrgA Impairs Hyphal Growth

**Figure 6:**
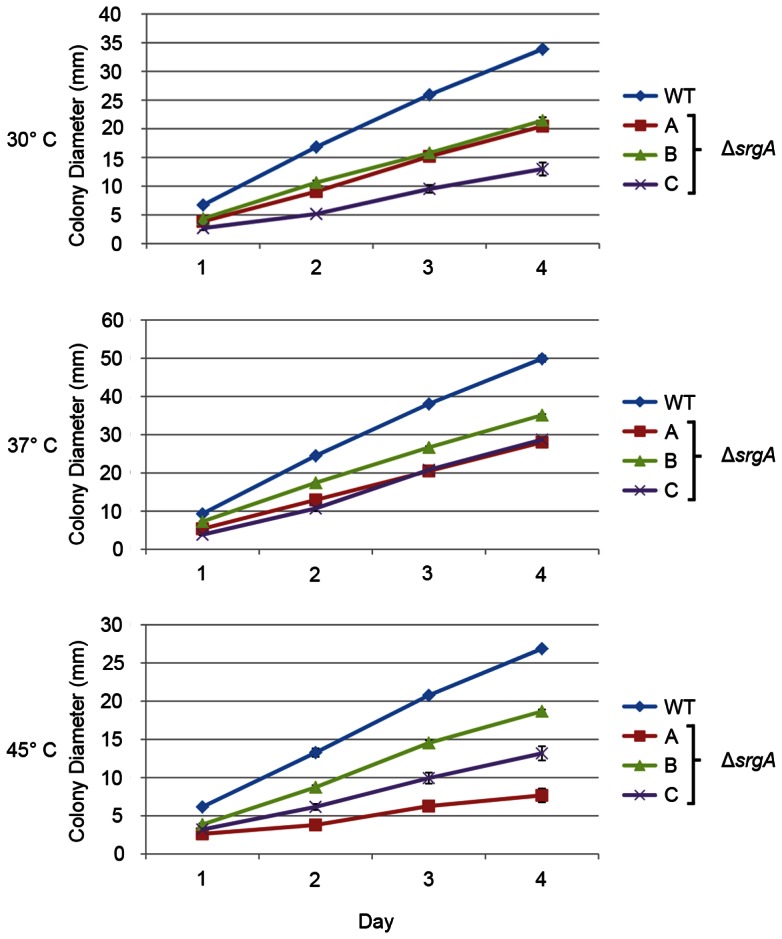
Loss of SrgA impairs hyphal growth. Equal numbers of conidia were plated on the center of a plate of solid AMM and colony diameter was measured every day during a four-day incubation period at the indicated temperatures. The experiment was performed in triplicate and the values represent the mean ± SEM.

In *A. niger*, the Δ*srgA* mutant displayed a two-fold increase in hyphal diameter, as well as unusual apical branching [17]. By contrast, hyphal morphology was normal in the *A. fumigatus* Δ*srgA* mutant, with no evidence of increased hyphal thickness or hyperbranching (data not shown). However, all three Δ*srgA* isolates were growth impaired at temperatures ranging from 30°C to 45°C. The extent of growth inhibition was variable between strains (Figure 6). For example, isolate C grew more slowly than the other two isolates at 30°C. However, at 37°C, isolate C grew at the same rate as isolate A, and only slightly slower than isolate B. At 45°C, all three strains grew at distinctly different rates, with isolate A being the most growth impaired. This phenotypic heterogeneity is consistent with the notion that each mutant harbors a different compensatory response to the loss of *srgA*, which impacts the ability of the organism to grow at different temperatures.

### Loss of SrgA Alters Susceptibility to ER stress

**Figure 7:**
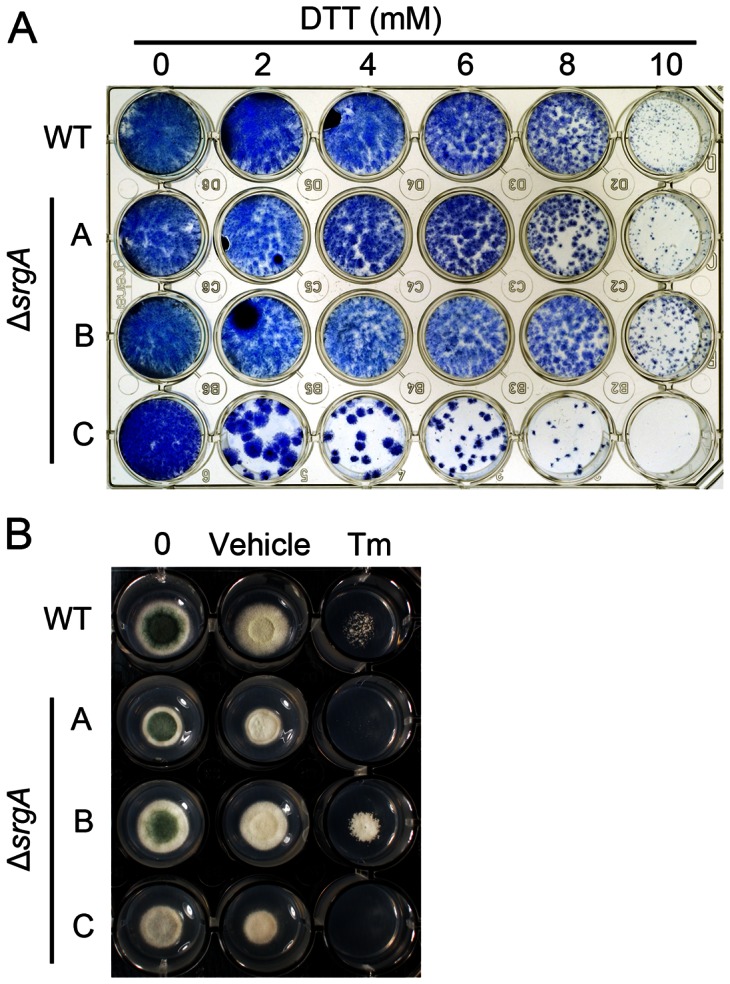
Sensitivity of Δ*srgA* to ER stress. A: Equal numbers of conidia were added to individual wells of a 24-well plate containing liquid AMM media and the indicated concentrations of dithiothreitol (DTT). Plates were incubated at 37°C for three days, after which the mycelial biomass that was adhered to the plate surface was stained with methylene blue and photographed. B: Equal numbers of conidia were inoculated onto solid AMM media containing either the vehicle control (DMSO) or 100 µg/ml tunicamycin (TM) and incubated for three days at 37°C.

**Figure 8:**
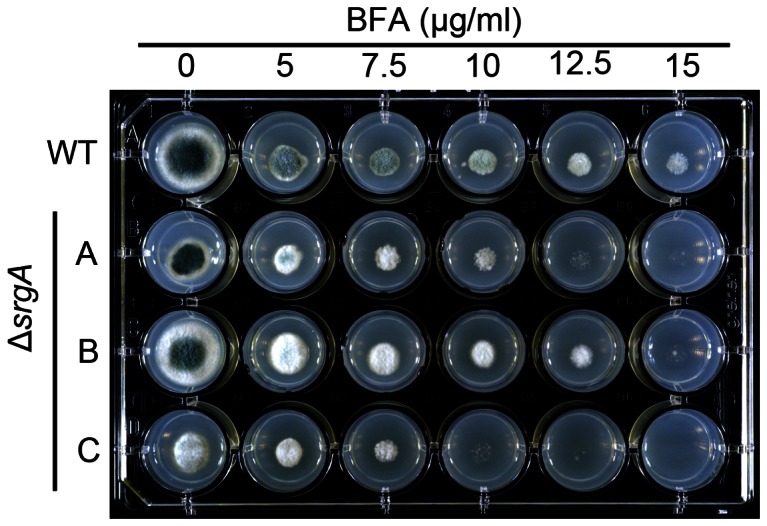
Sensitivity of Δ*srgA* to brefeldin A. Equal numbers of conidia were inoculated onto solid AMM media containing the indicated concentrations of brefeldin A (BFA) and incubated for three days at 37°C.

Mutations that adversely affect homeostasis of the secretory pathway are often associated with heightened sensitivity to agents that cause endoplasmic reticulum (ER) stress, such as dithiothreitol (DTT) and tunicamycin (TM) [22]. We found that loss of *srgA* was associated with hypersensitivity to DTT, but only in isolate C (Figure 7A). Similarly, isolates A and C were hypersensitive to TM, but isolate B was not (Figure 7B). However, all three isolates were hypersensitive to the ER stress-inducing agent brefeldin A (BFA) (Figure 8). It is likely that the shared hypersensitivity to BFA, relative to the more variable responses to DTT and TM, reflects differences in the mechanism by which each agent disrupts ER homeostasis. DT and TM induce generalized protein folding stress by interfering with disulfide bonds and N-linked glycosylation, respectively [22]. Thus, the divergent responses of the three Δ*srgA* isolates to DTT and TM may be due to the different compensatory changes that each isolate has undergone in order to mitigate the loss of SrgA function. By contrast, BFA disrupts vesicle trafficking between the ER and the Golgi [23]. Since Sec4 homologs also regulate vesicular trafficking [10], we speculate that BFA treatment in the absence of *srgA* is a more difficult obstacle to overcome by compensatory mechanisms because it induces a critical defect in vesicle trafficking homeostasis that is incompatible with growth.

### Loss of SrgA Alters Virulence

**Figure 9:**
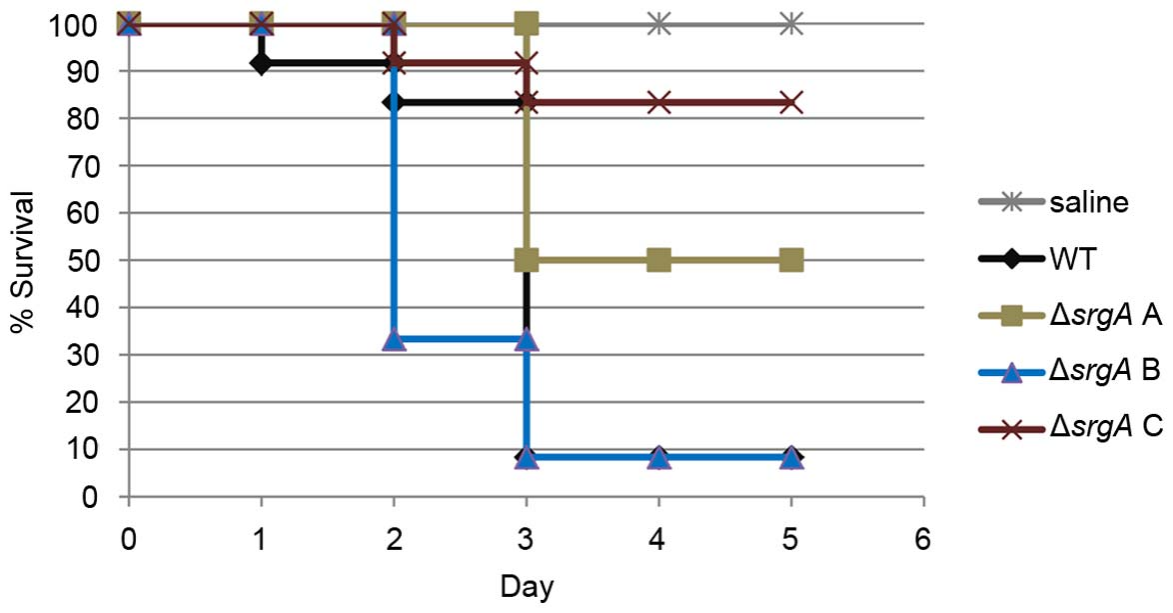
Analysis of Δ*srgA* virulence in an insect model of *A. fumigatus* infection. Groups of 12 *G. mellonella* larvae were infected with conidia from the indicated strains. Larvae were incubated at 37°C and mortality was monitored over a five day period. Kaplan-Meier survival curves were compared using a log-rank test, followed by a pairwise multiple comparison test (Holm-Sidak). The Δ*srgA* isolate C survival curve is statistically different from wt, but isolates A and B were indistinguishable from wt.

The virulence of each Δ*srgA* isolate was tested in a *Galleria mellonella* infection model. *G. mellonella* larvae do not have to be immunosuppressed in order to allow initiation of an *A. fumigatus* infection, which allows fungal pathogenesis to be studied in the context of an intact immune system [24], [25], [26]. Conidia were injected into the last pro-leg of sixth instar *G. mellonella* larvae and survival was monitored over five days. As shown in Figure 9, the Δ*srgA* isolate C had attenuated virulence relative to wt *A. fumigatus*. However, isolates A and B were statistically indistinguishable from wt, indicating there is also diversity among Δ*srgA* isolates with respect to virulence.

### Reproducibility of Phenotypic Heterogeneity Among Δ*srgA* Isolates

The discordant phenotypes observed between individual isolates of the *A. fumigatus* Δ*srgA* mutant suggested that the deletion of *srgA* selects for the acquisition of compensatory changes, such as second-site mutations. This complicates the interpretation of complementation studies, since the reconstitution of *srgA* into the three Δ*srgA* isolates is unlikely to correct the phenotypic heterogeneity because each isolate would still harbor unknown and potentially unique mutations in related pathways. When gene reconstitution is unsuitable for genetic deletion experiments, the isolation of a second, independently derived mutant strain can be used as alternative way to confirm a phenotype [27]. Thus, we performed a separate transformation experiment with the *srgA* knockout construct and obtained a second Δ*srgA* strain, designated Δ*srgA*-2. Similar to the original Δ*srgA* strain (Δ*srgA*-1), Δ*srgA*-2 revealed colony heterogeneity (Figure S1, A). Based on morphological similarities to the previous Δ*srgA*-1 isolates, three Δ*srgA*-2 isolates were selected (A, B, and C) and tested under *in vitro* growth conditions. All Δ*srgA*-2 isolates were growth impaired to the same extent as the Δ*srgA*-1 isolates (Figure S1, B). In addition, we identified increased sensitivity of all three Δ*srgA*-2 isolates to BFA compared to wt, but phenotypic divergence between the three Δ*srgA*-2 isolates in their sensitivity to DTT, similar to what was observed in the Δ*srgA*-1 isolates (Figure S1, C and D). The observation that phenotypic heterogeneity occurs in two independently isolated Δ*srgA* mutants suggests that loss of *srgA* is the predisposing factor for *A. fumigatus* to undergo additional alterations to mitigate the effects of *srgA* deficiency.

## Discussion

In this study we deleted the *A. fumigatus srgA* gene, encoding a Rab GTPase homologue that is closely related to Sec4. The most striking finding was that *srgA* deletion was associated with phenotypic heterogeneity, which was manifested by distinct colony morphologies and variable responses to both *in vitro* and *in vivo* stress conditions. Phenotypic variability was not observed in the corresponding mutant in *A. niger*, [17] suggesting fundamental differences between the two species with respect to the response to SrgA deficiency. This phenotypic variation was also not observed in the *A. fumigatus* parental strain used in this study, nor in other mutants that have been generated on the same genetic background [28], [29], [30], which implicates the loss of *srgA* as the predisposing factor for these diverse phenotypes. It is worth noting that the frequency of homologous targeting was very low for this gene; only two Δ*srgA* mutants were identified in a screen of approximately 100 transformants from two genetic backgrounds (*kuA* and CBS 144.89). This is consistent with the notion that the loss of *srgA* creates a severe phenotypic defect, possibly lethality, which selects for suppressor mutations to compensate for the defect. We speculate that one or more such mutations have occurred within each of the Δ*srgA* isolates, which improves the fitness of the fungus beyond that of the original Δ*srgA* strain. These could be multi-copy suppressors derived from other members of the Rab GTPase family, or mutations in genes in related pathways that can partially compensate for the absence of SrgA. Unfortunately, while genetic models to identify suppressor mutations are well established in yeast, and have been previously used to discover suppressors of Rab GTPase mutants [31], [32], [33], [34], [35], [36], [37], [38], such techniques are poorly developed in *A. fumigatus*. Therefore, secondary mutations that may be contributing to the phenotypic heterogeneity of the Δ*srgA* isolates remain to be identified.

Despite the heterogeneity among Δ*srgA* isolates, all of them shared the same phenotype of reduced radial growth rate and abnormal conidiation. This finding is consistent with the defects in polarized growth and sporulation reported for *srgA*-disruption mutants in *A. niger* [17]. Interestingly, only one of the three *A. fumigatus* Δ*srgA* isolates had attenuated virulence, making it unclear whether it is the loss of *srgA* or associated compensatory mutations that contribute to reduced pathogenicity in this model. However, since the three isolates grow at the same rate *in vitro*, the observed reduction in pathogenicity is not simply due to a slower growth rate. Rather, attenuated virulence correlated more closely with stress response: the Δ*srgA* isolates that exhibited a superior ability to adapt to *in vitro* stress showed wt virulence, whereas the isolate with the least resistance to *in vitro* stress had attenuated virulence.

The findings from the current study demonstrate that *A. fumigatus* is capable of surviving without SrgA-specific functions. However, the unexpected phenotypic heterogeneity that accompanies the loss of SrgA suggests that a variety of mechanisms are triggered to compensate for the absence of SrgA, some of which may be suppressor mutations. Future studies to elucidate these compensatory changes may provide important insight into networks that support homeostasis of the secretory pathway in this important fungal pathogen.

## Materials and Methods

### Culture Conditions

Strains used in this study are listed in Table 1. Conidia were harvested from strains grown on OSM plates [29] (*Aspergillus* minimal media (AMM) containing 10 mM ammonium tartrate and osmotically stabilized with 1.2 M sorbitol). Unless otherwise noted, all experiments were conducted at 37°C. For analysis of dithiothreitol (DTT) susceptibility, 5,000 conidia were inoculated into each well of a 24-well plate containing liquid AMM supplemented with increasing concentrations of DTT. Plates were incubated at 37°C for three days without shaking. The medium was then aspirated, and the hyphae adhering to the base of the well were stained with 0.5% (w/v) methylene blue for one hour at 37°C. After removing the methylene blue solution, the adherent hyphae were rinsed with sterile water and dried prior to photographing. Sensitivity to tunicamycin (100 µg/ml) and brefeldin A (5–15 µg/ml) was determined by spotting conidia into each well of a 24-well plate containing AMM with increasing concentrations of the compound and incubating for 2–3 days at 37°C.

**Table 1:** Strains used in this study.

Name	Description	Source
wt (AfS28)	Δ*akuA::ptrA*	S. Krappmann
Δ*srgA-1* and Δ*srgA-2*	(AfS28), Δ*akuA::ptrA*, Δ*srgA::ble*	This study
CBS144.89	Wild type	R. Cramer
GFP-SrgA	(CBS144.89), *gfp- srgA/ble*	This study

For analysis of hyphal growth, conidia were spot-plated onto the surface of a plate containing AMM agar and radial growth was monitored over a four-day incubation period at 37°C. The rate of radial growth was calculated as the colony diameter on day four minus the initial colony diameter after the first 24 hours of incubation divided by the incubation period.

### Analysis of Intracellular Localization by GFP-Tagging

PCR primers used to construct a GFP-srgA expression cassette are listed in Table S1. Total DNA was extracted from overnight cultures of wt *A. fumigatus* and *srgA* was PCR amplified using primers 824 and 825. The PCR product was then inserted into the *Nde*I and *Not*I sites of p538, a GFP-fusion cassette driven by the *Aspergillus nidulans gpdA* promoter [39], thus generating p626. Plasmid 626 was then ectopically introduced into the wt strain CBS144.89. The intracellular localization of the fusion protein was then determined by inoculating conidia from the GFP-SrgA *A. fumigatus* strain onto a glass coverslip submerged in liquid AMM and incubating overnight at 37°C. Coverslips, with adhered germlings on the surface, were then inverted and mounted on a glass slide. Images were acquired with a Zeiss LSM710 confocal with an Axio Observer Z1 set for GFP detection. Images of developing conidiophores were acquired using an Olympus IX71 inverted microscope set for GFP detection.

### Deletion of A. fumigatus srgA

The gene encoding *A. fumigatus* SrgA (AFUA_4G04810) was replaced with the phleomycin resistance gene using the split-marker method [40]. The first two-thirds of the phleomycin resistance cassette were amplified from pAN7-1 using primers 398 and 408, creating PCR product #1. The second two-thirds of phleomycin were then amplified with primers 409 and 410, creating PCR Product #2. The left arm of the *srgA* gene was amplified from wt DNA using primers 694 and 695, and the right arm was amplified with primers 696 and 697, generating PCR products #3 and #4, respectively. PCR products #1 and #3 were then combined in an overlap PCR reaction with primers 398 and 695 to generate PCR product #5 and PCR products #2 and #4 were combined in an overlap reaction with primers 696 and 410 to generate PCR product #6. PCR products #5 and #6 were then cloned into pCR-Blunt II-TOPO (Invitrogen) to create plasmids p599 and p600, respectively. The p599 and p600 plasmids were linearized with *Xho*I/*Bam*HI and *Eco*RI, respectively, and transformed into AfS28 (referred to here as wt) *A. fumigatus* protoplasts as previously described [39]. For each transformation, phleomycin-resistant colonies were plucked from the original selection plate and transferred to secondary plates containing phleomycin. Monoconidial strains were obtained after two passages, in which conidia were spread on selection-free media and individual colonies were isolated. Conidia from monoconidial colonies were then used to create the final 10% glycerol stocks.

### Analysis of Conidiophore Development

For analysis of conidiophore morphology, conidia were inoculated onto the edge of an OSM agar plug. A glass coverslip was placed on top of the plug and incubated for three days at 37°C. The coverslips were removed, mounted on a glass slide, and condiophores were observed using bright-field microscopy. For analysis of conidia morphology, wt and the Δ*srgA* isolates were incubated on OSM plates for ten days at 37°C in tissue culture flasks; the flasks were then removed and incubated at room-temperature (RT) for seven days (RT incubation facilitated the conidiation of Δ*srgA* isolate C). Conidia were then harvested from the plates and analyzed microscopically.

### *G. mellonella* Infection Model

*G. mellonella* larvae in the final instar stages were obtained from Vanderhorst, Inc (St. Marys, OH). Twelve larvae per group, weighing between 250–350 milligrams, were inoculated with conidia from either wt *A. fumigatus* or one of the Δ*srgA* isolates. Five microliters of a 1×10^8^ conidia/ml saline suspension (5×10^5^ conidia) were injected into the last left pro-leg of each larva using a Hamilton syringe (Hamilton Company, Nevada). Six larvae were included in a control group, with each larva receiving an inoculum of five microliters of saline. The larvae were placed in petri dishes and incubated in the dark at 37°C for five days. Larvae were examined daily and mortality was defined as lack of movement upon physical stimulation. Survival rates were recorded using a Kaplan-Meier survival curve and analyzed using a log-rank test, followed by a Holm-Sidak test for pairwise multiple comparisons (Sigma Stat 3.5).

## Supporting Information

Figure S1 **Phenotypic heterogeneity is a reproducible phenotype associated with** ***srgA*** **deletion.** A second transformation was performed in order to obtain another, independently isolated Δ*srgA* mutant (Δ*srgA*-2). A: The Δ*srgA-2* mutant showed the same colony heterogeneity as the original Δ*srgA* shown in Fig. 3. B: Three different isolates of Δ*srgA-2* were spotted onto AMM and incubated at 37°C for four days. Radial growth rate was determined by measuring colony diameter after the first 24 hours of incubation [*statistically significant by Student's T-test (p<0.001)]. C: Equal numbers of conidia were inoculated onto solid AMM media containing increasing concentrations of brefeldin A (BFA) and incubated for two days at 37°C. D: Equal numbers of conidia from the three isolates of Δ*srgA-2* were added to individual wells of a 24-well plate containing liquid AMM media and the indicated concentrations of dithiothreitol (DTT). Plates were incubated at 37°C for three days, after which the mycelial biomass that was adhered to the plate surface was stained with methylene blue and photographed.
(TIF)

Table S1 **PCR primers used in this study.** M13-derived sequences used for overlap PCR are underlined.
(DOCX)
