# Supplementary figures and images for: Deletion of the sec4 Homolog srgA from Aspergillus fumigatus Is Associated with an Impaired Stress Response, Attenuated Virulence and Phenotypic Heterogeneity
Source: PLoS One. 2013 Jun 13;8(6):e66741. doi: 10.1371/journal.pone.0066741 (PMC3681910; doi:10.1371/journal.pone.0066741)

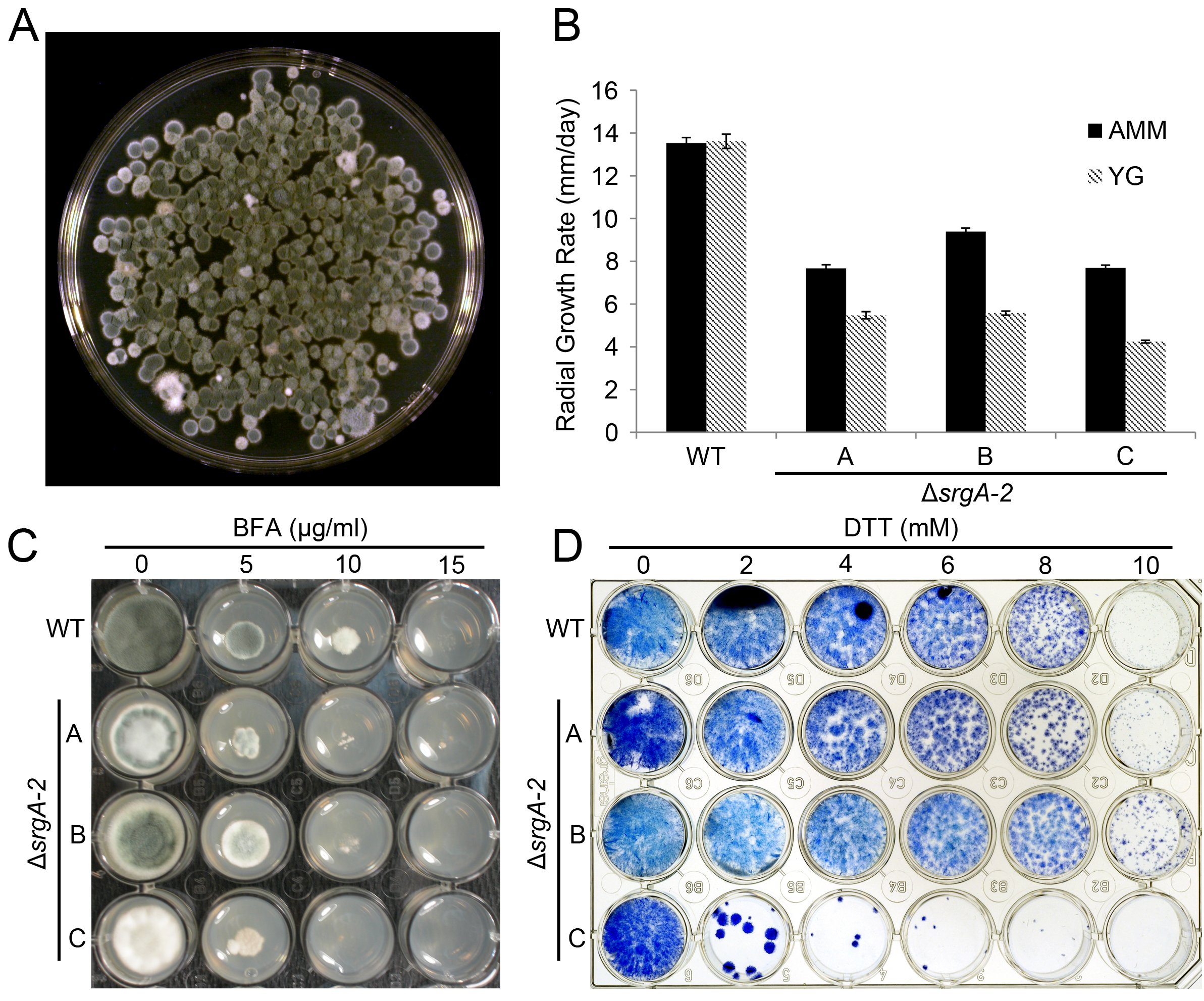

Supplement: Figure S1 — Phenotypic heterogeneity is a reproducible phenotype associated with srgA deletion. A second transformation was performed in order to obtain another, independently isolated ΔsrgA mutant (ΔsrgA-2). A: The ΔsrgA-2 mutant showed the same colony heterogeneity as the original ΔsrgA shown in Fig. 3. B: Three different isolates of ΔsrgA-2 were spotted onto AMM and incubated at 37°C for four days. Radial growth rate was determined by measuring colony diameter after the first 24 hours of incubation [*statistically significant by Student's T-test (p<0.001)]. C: Equal numbers of conidia were inoculated onto solid AMM media containing increasing concentrations of brefeldin A (BFA) and incubated for two days at 37°C. D: Equal numbers of conidia from the three isolates of ΔsrgA-2 were added to individual wells of a 24-well plate containing liquid AMM media and the indicated concentrations of dithiothreitol (DTT). Plates were incubated at 37°C for three days, after which the mycelial biomass that was adhered to the plate surface was stained with methylene blue and photographed. (TIF) [file pone.0066741.s001.tif]
