# Supplementary material for: Deletion of the sec4 Homolog srgA from Aspergillus fumigatus Is Associated with an Impaired Stress Response, Attenuated Virulence and Phenotypic Heterogeneity
Source: PLoS One. 2013 Jun 13;8(6):e66741. doi: 10.1371/journal.pone.0066741 (PMC3681910; doi:10.1371/journal.pone.0066741)
Supplement: Table S1 — PCR primers used in this study. M13-derived sequences used for overlap PCR are underlined. (DOCX) [file pone.0066741.s002.docx]

| **Primer** | **Gene** | **Sequence (5’-3’)** |
| --- | --- | --- |
| 398 | *ble* | cgccagggttttcccagtcacgacaagtggaaaggctggtgtgc |
| 408 | *ble* | TGCTCGCCGATCTCGGTCAT |
| 409 | *ble* | AGCGGATAACAATTTCACACAGGATTAAAGCCTTCGAGCGTCC |
| 410 | *ble* | GACAAGGTCGTTGCGTCAGTC |
| 694 | *srgA* | AGCATCCATTAGAGACAGCC |
| 695 | *srgA* | GTCGTGACTGGGAAAACCCTGGCGTCAAAAGAGCCGGTACTTGG |
| 696 | *srgA* | TCCTGTGTGAAATTGTTATCCGCTGTTGTTATCCGCTGTTGTTAGGCATGCGCTTTC |
| 697 | *srgA* | GTCCTCGAAGTTTGCACAAC |
| 758 | *srgA* | CACTTTGGTGGCCATAGATC |
| 759 | *srgA* | CCGATCCGTGATGGTCTATA |
| 824 | *srgA* | GGAATTCCATATGGCCGGCACAAGAAATTA |
| 825 | *srgA* | ATAAGAATGCGGCCGCCTAACAACATTTACCGCCAG |
